# The Healing Effect of Photobiomodulation on Venous Leg Ulcers: A Systematic Review and Meta‐Analysis

**DOI:** 10.1111/wrr.70144

**Published:** 2026-03-26

**Authors:** Aqella Rasul, Frederik Plum, Katrine Elisabeth Karmisholt, Klaus Kirketerp‐Møller

**Affiliations:** ^1^ Copenhagen Wound Healing Center, Department of Dermatology, Bispebjerg Hospital Copenhagen Denmark; ^2^ Department of Dermatology, Bispebjerg Hospital Copenhagen Denmark; ^3^ Department of Clinical Medicine University of Copenhagen Copenhagen Denmark

**Keywords:** low‐level‐laser therapy, meta‐analysis, photobiomodulation, phototherapy, systematic review, venous leg ulcers, wound healing

## Abstract

Venous leg ulcers are common, costly and often slow to heal despite standard management. Photobiomodulation has been proposed as an adjuvant therapy to enhance wound repair. This systematic review and meta‐analysis aimed to evaluate the effect of photobiomodulation on healing outcomes in venous leg ulcers. A comprehensive search of PubMed, Embase, Web of Science and Cochrane Library (1990–2024) identified randomised controlled trials evaluating photobiomodulation in adults with venous leg ulcers. Two reviewers independently screened studies (*n* = 3824) and assessed eligibility according to predefined inclusion and exclusion criteria (*n* = 34). Data were extracted from the included studies (*n* = 11), and the risk of bias was assessed using the Cochrane RoB 2 tool. A random‐effects meta‐analysis was conducted for the absolute change in ulcer area. Eleven trials were included, comprising 615 randomised patients, where 340 had venous leg ulcers. Patients who completed follow‐up contributed to the healing outcomes (intervention *n* = 169, controls *n* = 167). All studies were evaluated to have some or high concerns for risk of bias. Four trials contributed data to the meta‐analysis showing that photobiomodulation did not significantly reduce ulcer area compared with controls (Mean Difference 3.77 cm^2^, 95% CI −4.45 to 11.99; *p* = 0.37) and heterogeneity was substantial (*τ* ^2^ = 65.37; *I* ^2^ = 96%). Current evidence does not demonstrate a statistically significant benefit of photobiomodulation on venous leg ulcer healing. High heterogeneity, small sample sizes and methodological limitations reduce confidence in the pooled estimate. Standards for photobiomodulation therapy in venous leg ulcers are still missing, and well‐designed RCTs are needed to clarify the therapeutic potential of photobiomodulation.

**Abbreviations**
ATP: adenosine triphosphate
LED: light‐emitting diode
LLLT: low‐level light therapy
MeSH: Medical Subject Headings
PBM: photobiomodulation
PRISMA: Preferred Reporting Items for Systematic reviews and Meta‐Analyses
PRISMA‐P: Preferred Reporting Items for Systematic Review and Meta ‐Analysis Protocols
RCT: randomised clinical trials
ROS: reactive oxygen species
TGF‐β1: transforming growth factor beta 1
VAS: visual analogue score
VEGF: vascular endothelial growth factor
VLU: venous leg ulcers

## 1 Introduction

Photobiomodulation using red and near‐infrared light has been proposed as an adjuvant therapy to accelerate the healing of venous leg ulcers. To this date, however, recommendations based on the highest level of evidence are still missing.

The management of leg ulcers is a substantial socio‐economic burden, accounting for up to 1% of total healthcare expenditure in European countries [1, 2]. The prevalence of lower limb ulcers varies across countries. In 2003, the prevalence of foot and leg ulcers in northern Europe and Australia ranged between 0.06%–3.6% [3]. In contrast, data from the United Kingdom reported prevalences of 0.045% for leg ulcers alone, of which an estimated 43% were of venous origin [4]. Venous leg ulcers represent the end‐stage of chronic venous disease driven by progressive venous hypertension. Venous hypertension arises from vein valve incompetence, reflux and altered vasomotor tone but is also influenced by functional factors such as oedema, weak calf muscle and obesity [5]. Sustained venous hypertension induces endothelial leakage of inflammatory cells and mediators. The result is oedema, inflammation, fibrosis, pigmentation and calcification that leads to the disintegration of the skin and thus the formation of ulcers [6]. Compression alleviates venous hypertension, and is considered a cornerstone in venous ulcer therapy; nonetheless, healing is not always achieved with this regimen, and 10‐year recurrence rates are 60%–70% [7, 8]. In a database study of 777 venous leg ulcer patients, 42.2% healed within 3 months, and 48.6% within 6 months, suggesting that some wounds do not respond to standard therapy and may need more advanced treatments [9].

Hence, adjuvant therapies such as photobiomodulation are being explored. Photobiomodulation refers to the non‐thermal, non‐ionising light therapy in the visible and near‐infrared spectrum. When photons are absorbed in the cellular chromophores, they will initiate photophysical and photochemical reactions in these cellular structures [10]. The most studied target is Cytochrome C in mitochondria, when red and near‐infrared light reach the chromophore. Absorption of the photon induces dissociation of an inhibiting nitric oxide molecule from Cytochrome C, augmenting respiratory chain activity and production of adenosine triphosphate (ATP) and reactive oxygen species (ROS) [11]. ATP and ROS induce cellular proliferation, migration and the expression of transcription factors and growth factors, including transforming growth factor beta 1 (TGF‐β1) [12]. In vitro and in animal studies have demonstrated that red and near‐infrared photobiomodulation increases wound healing [13, 14, 15, 16]. Clinical systematic reviews on diabetic and pressure ulcers using photobiomodulation treatment demonstrate significant reduction of ulcer areas and accelerated healing rates [17, 18]. A prior attempt to synthesise systematic evidence on venous leg ulcers was made by Flemming et al. in 1999; however, this review was withdrawn again in 2014 [19]. Since then, mechanistic evidence has expanded substantially, and consumer‐accessible LEDs and low‐level laser therapy devices have become widely available ‐ creating an urgent need for critical appraisal of the clinical evidence base.

The aim of this systematic review is to evaluate the effect of photobiomodulation therapy on wound healing in patients with venous leg ulcers. The primary outcome is ulcer healing quantified as absolute or relative change in ulcer size or in time‐to‐heal. Secondary outcomes were to investigate quality of life, pain and adverse events of the photobiomodulation treatment.

## 2 Method

### 2.1 Eligibility

This study included randomised controlled trials published in English between January 1990 and February 2025 that evaluated photobiomodulation therapy for venous leg ulcers. Photobiomodulation modalities, including low‐level laser therapy and LED‐based devices, were eligible. Adults with venous leg ulcers of any size and duration were eligible. We accepted the authors' definition of venous leg ulcers irrespective of diagnostic method. Standard care was permitted in both intervention and comparator arms. Eligible outcome measures comprised absolute or relative change in ulcer area or time‐to‐healing. Comparator groups were placebo/sham when available. If no placebo arm existed, standard care alone was used as the control. In vitro and animal studies, ultraviolet light applications and photodynamic therapy were excluded.

### 2.2 Search Strategy

**FIGURE 1:**
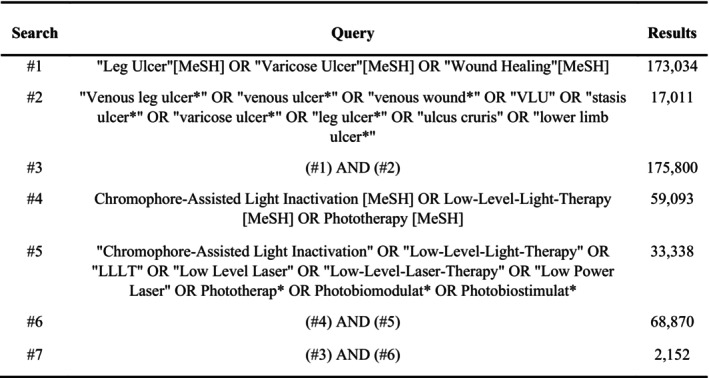
Search query in PubMed.

Prior to the screening start, a study protocol was written, following the PRISMA‐P 2015 guidelines [20] and was registered at PROSPERO (Centre for Reviews and Dissemination, University of York, York, UK) (CRD42024536451). Results of this study are reported in accordance with the PRISMA 2020 guidelines [21]. Preliminary searches investigating relevant key words and MeSH terms were performed to create the search query in February 2025. The search query was built up from the PICO‐model [22], consisting of aspects with relevant key words and MeSH terms (Figure 1). The final search was approved with respect to methodology and coherence by a librarian from the Royal University Library of Copenhagen.

### 2.3 Screening

The search was performed on 20th February 2025. Titles and abstracts were exported from the following databases: PubMed, Embase, Web of Science and Cochrane Library. References were imported into the reference tool COVIDENCE [23] that automatically removed duplicates. Screening of titles and abstracts was performed by the two reviewers A.R. and F.P., independently. Disagreements were resolved by the third reviewer K.K.M. If the title and abstract met the inclusion criteria, a full‐text review for eligibility was performed by A.R. and F.P. independently. No additional records were identified through reference lists or with citation tracking.

### 2.4 Data Extraction

Data extraction was performed by two reviewers A.R. and F.P., independently. Disagreements were resolved by discussion. The following data were extracted from the articles: Authors, publication year, country, study characteristics, patient characteristics, type of lighting device, wavelength, irradiance, treatment time, number of treatments, wound sizes, wound healing rates and pain scores.

### 2.5 Risk of Bias

The 2019 Cochrane Risk of Bias 2 Tool for RCT's was performed by two reviewers independently to assess risk of bias; disagreement was thereafter resolved in consensus [24]. The assessment includes the following five domains of bias: (1) the randomisation process; (2) deviations from intended intervention; (3) missing outcome data; (4) measurement of the outcome; and (5) selection of the reported result. Each domain was rated as either low risk of bias, some concerns, or high risk of bias. An overall judgement was made based on the judgement from the five domains according to the Cochrane Risk of Bias 2 Tool [24].

### 2.6 Data Synthesis

When multiple healing endpoints were reported within a trial, the following hierarchy was used: absolute change in ulcer area, then relative change in ulcer area, then time‐to‐healing. Trial characteristics, intervention parameters, wound‐healing outcomes and standard‐of‐care interventions were tabulated to assess comparability of trials for potential quantitative synthesis. When available, standard deviations, standard errors, or variances were extracted directly. If summary variances were not reported, standard deviations were derived from reported *p*‐values when these were provided with sufficient details. No further imputation was made concerning variances, and reported medians were not converted.

## 3 Results

### 3.1 Study Selection

**FIGURE 2:**
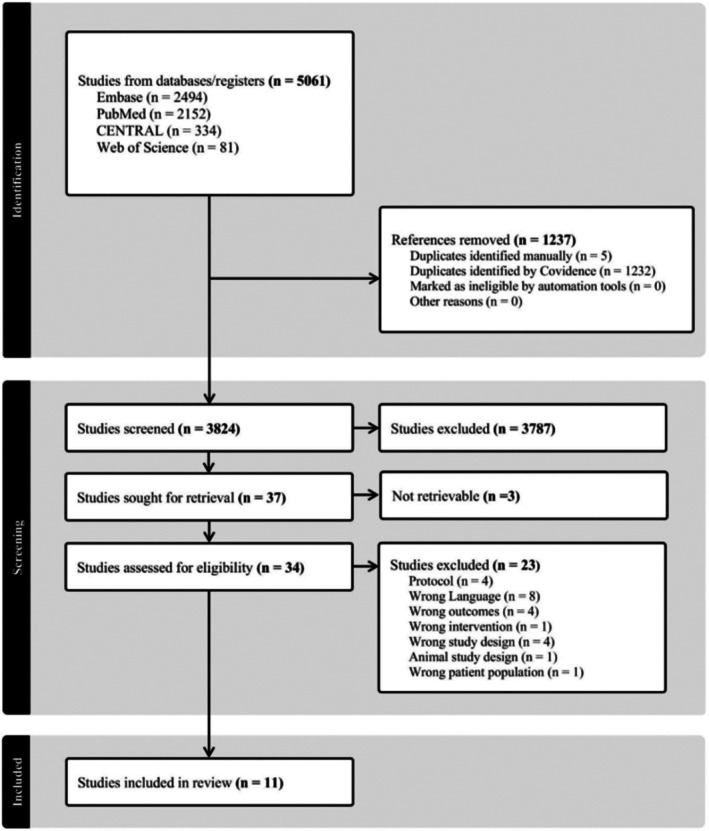
PRISMA flowchart showing the selection of studies included in this review. RCT, randomised controlled trials.

Searches in literature databases yielded 5061 records and were imported into Covidence for screening. Covidence identified 1232 duplicates, and five further duplicates were removed manually during screening, leaving 3824 records for title and abstract screening of inclusion criteria. Full text review was made on 37 records, and 26 records did not meet exclusion criteria, resulting in 11 studies fulfilling both inclusion and exclusion criteria (Figure 2).

### 3.2 Study Characteristics

Eleven randomised controlled trials, published between 1991 and 2024, were included [25, 26, 27, 28, 29, 30, 31, 32, 33, 34, 35]. Seven trials were double‐blinded, one was single‐blinded and three used an open‐label design. Placebo comparators were used in nine trials as either a sham or an inactive device. Two trials used standard of care as the control [29, 30]. Across all studies, 615 patients were randomised with mean ages from 60.5 to 73.5. One trial enrolled 305 patients with venous leg ulcers; however, only 21 patients received light photobiomodulation [29]. In total, 340 venous leg ulcers were included, and 169 were treated with light intervention as intended, and 167 were allocated as controls (Table 1). Kopera et al. reported attrition of four patients in the placebo arm and three in the light arm. One study further reported two dropouts [32] and two studies reported one dropout each prior to follow‐up [26, 35]. Four trials explicitly declared no conflicts of interest [27, 28, 30, 33], whereas the remaining trials did not report conflicts of interest. No Adverse Events or side effects were reported in the included studies.

**TABLE 1:** Study characteristics.

Author, Year	Design	Blinding	Control	Primary outcome	Follow up, weeks	Participants, n	Venous leg ulcers, n	Light group, n	Control group, n	Mean age, years
Malm et al. (1991)	RCT	Double	Placebo	Time to heal	12	42	42	21	21	60.5
Gupta et al. (1998)	RCT	Double	Placebo	Change in ulcer area and rate of healing	10	9	12	6	5^a^	62.9
Franek et al. (2002)	RCT	Open	Placebo	Change in ulcer area and volume	4.75	65	43	21	22	65.5
Lagan et al. (2002)	RCT	Double	Placebo	Change in ulcer area	12	15	16	8	7*	69.9
Kopera et al. (2005)	RCT	Double	Placebo	Reduction in wound size	13	44	28	14^a^	14^a^	66.6
Caetano et al. (2009)	RCT	Double	Placebo	Ulcer healing rate	13	20	25	14	11	NA
Leclére et al. (2010)	RCT	Open	Standard of care	Percentage change in area	9	34	34	18	16	73.5
Taradaj et al. (2012)	RCT	Open	Standard of care	Rate of healing	7	305	48	21	27	61.1
Siqueira et al. (2014)	RCT	Single	Placebo	Change in ulcer area, healing time and TNF –α levels.	25–30	17	28	13^a^	13^a^	60,11
Vitse et al. (2017)	RCT	Double	Placebo	Complete wound closure	12	24	24	13	11	67.0
Pasek et al. (2024)	RCT	Double	Placebo	Change in surface area	10	40	40	20	20	68.0

^a^ Dropout.

### 3.3 Study Interventions

The GaAs/GaAlAs laser modality was used in four trials [25, 29, 34, 35], and InGaAs in one [30], wavelengths 810–980 nm; total fluences 1.96–90 J/cm^2^. Five trials used LED, LEP or diode emitters 625–685 nm; 2.95–4 J/cm^2^ [26, 28, 31, 32, 33], and one trial used polychromatic light 500–2500 nm; 30 J/cm^2^ [27]. Follow‐up duration ranged from 4.75 weeks [25] to 30 weeks [32], with seven trials reporting follow‐up of 10–13 weeks. Treatment regimens ranged from one to three sessions per week in seven trials, five to six sessions per week in three trials, and three to seven sessions per week in one trial (Table 2).

**TABLE 2:** Intervention characteristics.

Author, Year	Device	Manufacturer	Wavelength	Irradiance	Dose	Duration	Per week
Malm et al. (1991)	GaAs laser	Irradia	904 nm	4 mW	1.96 J/cm^2^	10 min	2
Gupta et al. (1998)	LEPT laser	International Medical Instruments Inc., Ontario, Canada	660 nm	6 mW	4 J/cm^2^	30 s	3
Franek et al. (2002)	GaA1As laser	CTL‐1106MX, High Power Devices Inc., USA	810 nm	65 mW	4 J/cm^2^	Time related to wound size	5
Lagan et al. (2002)	GaA1As laser	Biotherapy 3ML system. Omega Laser Systems, West Sussex U.K	660–950 nm	532 mW	12 J/cm^2^	NA	1
Kopera et al. (2005)	LED	Hermann Heltschl, Schlüesselberg, Austria	685 nm	200 mW	4 J/cm^2^	6–18 min	3–7
Caetano et al. (2009)	LED	Dynatron Solaris 705, Dynatronics Corporation, Salt Lake City, UT USA	660 nm and 890 nm	100 mW	3 J/cm^2^	30 s per 5 cm^2^	2
Leclére et al. (2010)	inGasAs laser	Pharaon, Osyris, Hellennes, France	980 nm	15 W	90 J/cm^2^	9.8 min	1
Taradaj et al. (2012)	GaA1As Laser	CTL1106MX Elektronika Elektromedycyna, Otwock Poland	810 nm	65 mW	4 J/cm^2^	Time related to wound size	6
Siqueira et al. (2014)	LED	Laboratory of Optics and Optic Electronics, Universidade Estadual de Londrina, Paraná, Brazil	625 nm	25 mW	4 J/cm^2^	2.40 min per 1 cm^2^	1
Vitse et al. (2017)	Diode laser	Erchonia ML‐Scanner	635 nm	2.46 × 10^−3^ W/cm^2^	2.95 J/cm^2^	20 min	2
Pasek et al. (2024)	Polychromatic polarised, low‐energy light	Solaris, Medicolox, Poland	500–2500 nm	50 mW/cm^2^	30 J/cm^2^	16 min	5

Abbreviations: LED, light‐emission diode; n, number; NA, not available; RCT, randomised controlled trials.

### 3.4 Standard of Care

Standard of care was delivered alongside both light and placebo interventions, and therefore varied with the frequency of the intervention (Table S1). Ulcer dressing changes between treatment visits were not described. Compression therapy was reported in eight of the 11 trials; four specified compression pressure (mmHg), whereas four did not. Three trials did not report whether compression therapy was used. Debridement was performed in six of the 11 trials. Five reported physical debridement, and one used enzymatic debridement. Systemic medical therapy for venous disease was used in two trials. One used micronised purified flavonoid fraction (MPFF) only; the other permitted MPFF, sulodexide, pentoxifylline, or acetylsalicylic acid. Dressings varied considerably across trials. Antibacterial or antiseptic agents were used in three trials (potassium permanganate baths, octenisept disinfectant and silver sulphadiazine 1% cream). Franek et al. used potassium permanganate baths followed by topical compresses with colistin, fibrolan, chloramphenicol, or gentamicin at the investigator's discretion.

### 3.5 Wound Healing Outcomes

Wound‐healing endpoints were reported, or could be calculated, for absolute change in nine trials and relative change in 10 trials. One study reported time‐to‐healing only. Raw individual‐patient data were unavailable, preventing standard deviation values from being directly imputed. When sufficient statistical details were reported, standard deviations were derived from reported *p*‐values, provided these were reported (Table 3). The crude mean of absolute change in ulcer area in the light groups was −6.13 ± 5.56 cm^2^ per study (range 0.00 to −19.3 cm^2^), compared with −2.44 ± 5.81 cm^2^ in controls (range + 13.0 to −6.7 cm^2^). When adjusted for follow‐up duration, this corresponded to mean daily reductions of 0.087 ± 0.080 cm^2^/day in light groups and 0.056 ± 0.059 cm^2^/day in controls. The mean relative change in ulcer area was −4.10% ± 2.21% per week with light therapy versus −3.85 ± 3.64% per week in controls. Marked baseline imbalances were noted; for instance, Caetano et al. reported ulcer areas of 21.0 cm^2^ at baseline in the intervention group versus 15.0 cm^2^ in controls. In Kopera et al., ulcers measured 4.5 cm^2^ versus 8.6 cm^2^, respectively; in this trial, data were extracted from a figure that presented no healing ulcers in the intervention group (0.00%). Gupta et al. likewise reported larger ulcers in the intervention group (40 cm^2^ vs. 20 cm^2^), but observed a 47.49% reduction with light therapy versus 7.34% in controls. Finally, Siqueira et al. observed a mean 75.00% reduction in the light group compared with an 85.00% increase in controls. Two studies reported pain scores [28, 35] using the Visual Analogue Score (VAS). Vitse et al. reported a significant decrease in pain in the test group 4 weeks after treatment, but after 12 weeks, a significant reduction was present in both test and placebo groups. Lagan et al. reported a statistically insignificant decrease in pain in both groups from baseline to follow‐up.

**TABLE 3:** Specific wound outcomes.

Author, Year	Group	Absolute change in ulcer area, cm^2^	Absolute change in ulcer area, cm^2^/week	Absolute change in ulcer area, cm^2^/day	Relative change in ulcer area baseline to follow up, %	Relative change in ulcer area, %, per week	P‐value on absolute difference baseline to follow up	P‐value on absolute difference between groups	P‐value on relative difference between groups
Gupta et al. (1998)	Light	19.3	1.930	0.276	−47.49	−4.749	NA	0.0002	NA
	Control	1.47	0.147	0.021	−7.34	−0.734	NA		
Franek et al. (2002)	Light	4.25	0.895	0.128	−26.97	−5.678	0.005	> 0.05	NA
	Control	5.21	1.097	0.157	−39.32	−8.278	0.003		
Lagan et al. (2002)	Light	NA	NA	0.000	−27.50	−2.292	NA	NA	0.14
	Control	NA	NA	0.000	−22.50	−1.875	NA		
Kopera et al. (2005)	Light	0.00	0.000	0.000	0.00	0.000	0.683	NA	NA
	Control	6.10	0.469	0.067	−70.93	−5.456	0.011		
Caetano et al. (2009)	Light	10.95	0.842	0.120	−50.00	−3.846	NA	NA	< 0.01
	Control	6.08	0.468	0.067	−40.00	−3.077	NA		
Leclére et al. (2010)	Light	2.10	0.233	0.033	−74.20	−8.244	NA	NA	0.60
	Control	3.16	0.351	0.050	−94.30	−10.478	NA		
Taradaj et al. (2012)	Light	4.99	0.713	0.102	−27.85	−3.979	NA	NA	> 0.05
	Control	5.42	0.774	0.111	−26.88	−3.840	NA		
Siqueira et al. (2014)	Light	6.00	0.200	0.029	−75.00	−2.500	NA	NA	> 0.05
	Control	−13.00	−0.433	−0.062	+85.71	2.857	NA		
Vitse et al. (2017)	Light	6.27	0.523	0.075	−77.10	−6.425	< 0.0001	> 0.8	NA
	Control	6.72	0.560	0.080	−69.20	−5.767	0.0013		
Pasek et al. (2024)	Light	1.35	0.135	0.019	−33.05	−3.305	< 0.001	NA	< 0.001
	Control	0.80	0.080	0.011	−18.99	−1.899	< 0.001		

### 3.6 Risk of Bias Assessment

**FIGURE 3:**
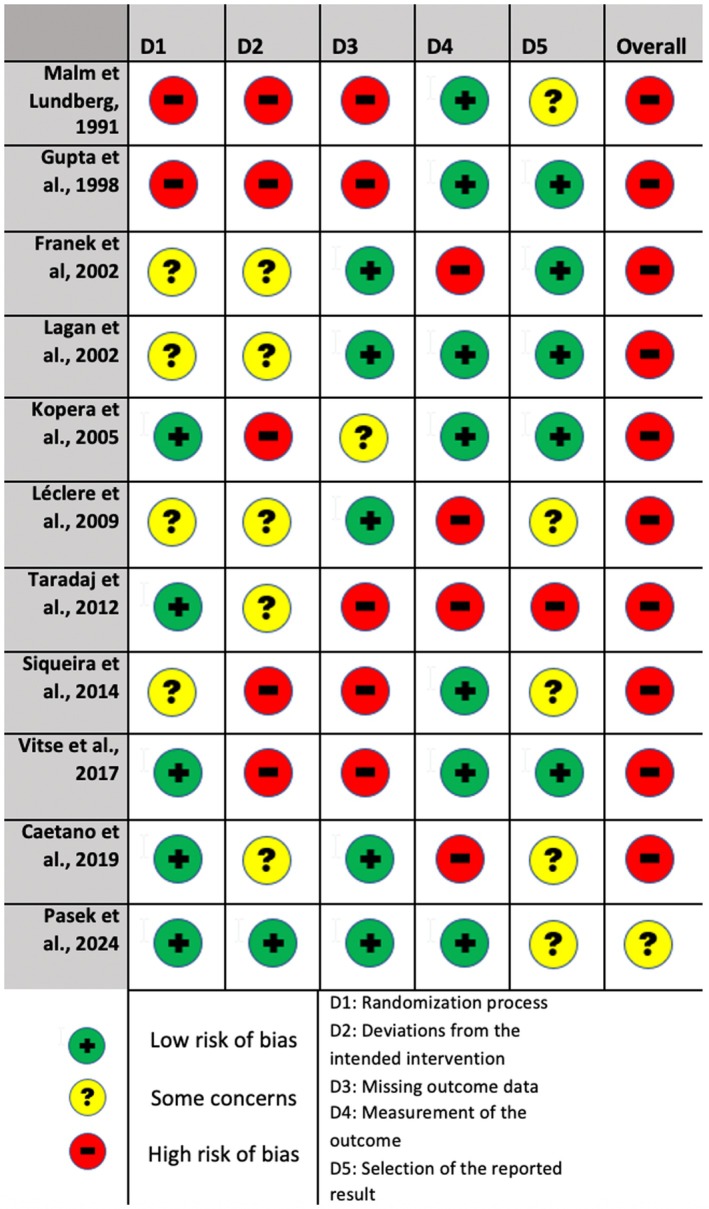
Risk of bias judgement for each of the included studies.

All 11 trials were assessed using the Cochrane Risk of Bias 2 tool. Ten trials were judged to be at high risk of bias (Figure 3), and one trial had 'some concerns'. Domain 1 (randomisation process) was commonly rated as 'some concerns' because randomisation procedures were insufficiently described. High risk of bias was most prominent in Domain 2 (deviations from intended interventions) and Domain 3 (missing outcome data). In Domain 3, this was mainly due to the absence of appropriate statistical handling of attrition [26, 31, 32]. For Domain 4 (measurement of the outcome), most trials used planimetric assessments from photographs or moulds; insufficient description of blinding of outcome assessment resulted in ‘high risk of bias’. In Domain 5 (selection of the reported result), most trials were judged ‘low risk’ or ‘some concerns’, and ethical approval was generally considered sufficient to mitigate major concerns relating to selective reporting.

### 3.7 Meta Analysis

**FIGURE 4:**
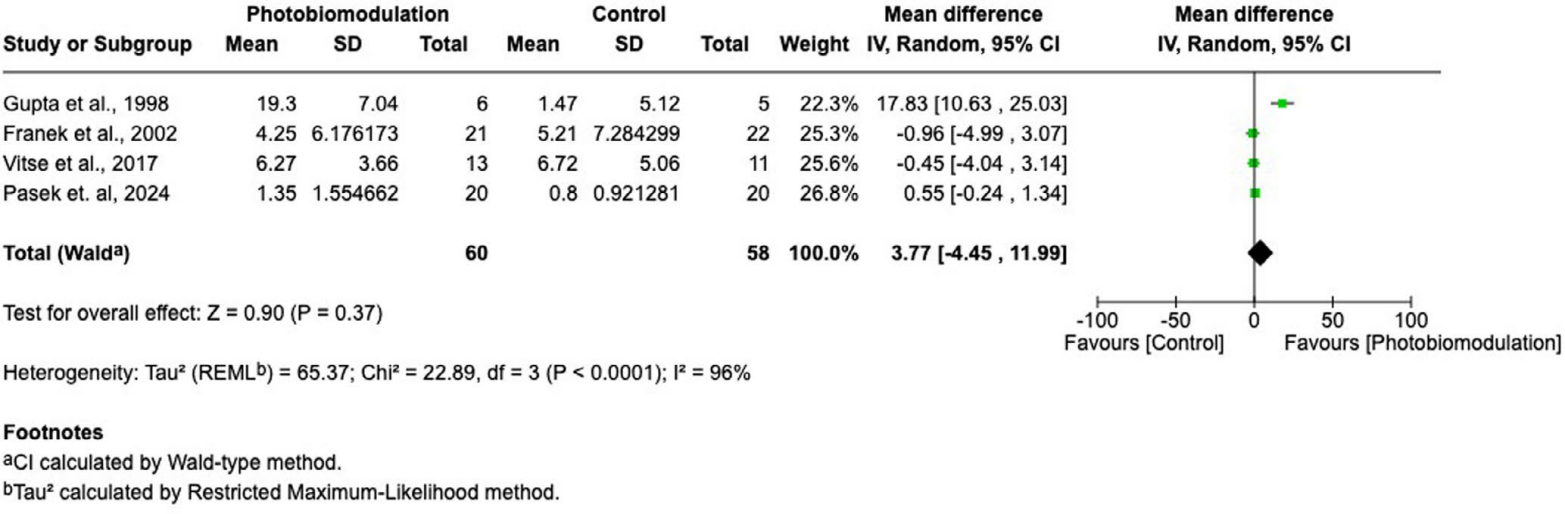
Meta‐analysis for change in ulcer area. CI, confidence interval; IV, invers variance; MD, mean difference; SE, standard error.

Suitable effect measures for meta‐analysis (effect estimate with corresponding standard deviation, standard error, or variance) were available only for absolute change in ulcer area, and only in four of the 11 trials [25, 26, 27, 28]. Treatment frequency ranged from two to seven sessions per week. One trial used infrared wavelengths at 4 J/cm^2^ [25], two used red wavelengths at 2.95–4 J/cm^2^ [26, 28] and one used a polychromatic light source (30 J/cm^2^) [27]. Compared with the control group, the photobiomodulation group had an absolute change in ulcer area of 3.77 cm^2^ 95% CI (−4.45; 11.99) and *p* = 0.37 (Figure 4). The random effect model was chosen because of the high heterogeneity between the studies (τ^2^ = 65.37, I^2^ = 96%, *p* < 0.00001).

## 4 Discussion

This primary outcome of this systematic review was to investigate changes in ulcer area between venous leg ulcers receiving photobiomodulation treatment and controls. Eleven randomised controlled trials were included, of which four were eligible for meta‐analysis. Meta‐analysis revealed a reduction in ulcer area of 3.77 cm^2^ when venous leg ulcers are treated with photobiomodulation and compared to controls; however, this was not statistically significant. The between‐study variance of 65.37 is high, and an indication of substantial variation, but also heterogeneity was high (96%), suggesting that the variance is not caused by random chance. Extraction of wound healing outcomes from all studies was not without challenges: baseline and follow‐up measures were often left out, medians were used instead of means, and statistical significance was not consistently reported. All but one trial was deemed to have a high risk of bias when assessed with the ROB. Deviations were most prominent when the intended interventions and the handling of missing outcomes were assessed. These circumstances suggest the results of the meta‐analysis should be interpreted with caution.

In order to elucidate heterogeneities, we compared the standard of care protocols between the studies. For instance, Gupta did not apply compression therapy, and Franek used potassium permanganate baths followed by various topical antibiotic dressings. Also, Gupta was included even though substantial baseline ulcer‐area imbalances were favouring the healing outcome. From a practical point of view, wound care handling with respect to products, contact layers, dressings and compression can vary substantially across countries—therefore also between studies. In wound healing studies, this constitutes an obvious ethical problem as the standard of care in wound healing studies should not be of less quality than otherwise offered, which may explain the use of oral medicine, antibiotic dressings and treatment at the investigator's discretion in these reported trials.

There is no consensus or standard treatment regimen for photobiomodulation therapy on venous leg ulcers [36]. This adds another level of complexity to the pooling of the extracted data. It is an example of a general problem regarding the photobiomodulation interventions in the literature, as treatment parameters often vary considerably across studies. However, to exert photobiomodulation effects, both lasers (collimated and coherent light), LED (non‐coherent light) and polychromatic light are generally accepted light sources [37]. The coherent and non‐coherent light both offer comparable photobiomodulation effects in expert opinions [11]. In addition, we chose to include near‐infrared and red wavelengths as the mode of action is believed to be caused by activation of the cytochrome c oxidase [38, 39]. Photobiomodulation is proposed to increase the healing rate of venous leg ulcers by activating cellular pathways that lead to an increase in cell proliferation, angiogenesis and reduction of inflammation [40]. In vitro studies on keratinocytes show that photobiomodulation at 661 nm enhances both the proliferation and migration [41]. Another explanation for this comes from Martignago et al. [42] who observed that fibroblasts exposed to Gallium‐Arsenide laser (904 nm) increased the expression of the gene for vascular endothelial growth factor (VEGF) together with collagen gene type 1 alpha 1. VEGF is known as an important and potent pro‐angiogenic factor [43], and collagen type 1 is a protein found in the extracellular matrix in the skin and plays an important role in tissue repair [42]. In 2021, Huang et al. performed a systematic review and meta‐analysis of low‐level light laser therapy in the healing of diabetic foot ulcers [44]. This study showed that LLLT significantly reduced the ulcer area measured as a percentage reduction in patients with diabetic foot ulcers. However, like the results presented here, a high level of heterogeneity was detected. A similar effect of LLLT on diabetic foot ulcers was observed in another systematic review by Santos et al. [45]. Both reviews included a few studies with small sample sizes in the meta‐analysis.

As secondary outcomes, this review wanted to investigate quality of life, pain and adverse events. None of the studies reported on the quality of life. Two studies reported pain scores [28, 35] using the Visual Analogue Score (VAS). Vitse et al. reported that pain decreased significantly in both the test and placebo groups from baseline to 12 weeks. Interestingly, a decrease after 4 weeks post‐treatment was only significant for the test group. Lagan et al. reported a statistically insignificant decrease in pain in both groups from baseline to follow‐up. These results are in line with Santos et al. [45]. who reported mixed effects of LLLT on pain reduction in a systematic review. Since only two studies reported pain, and one of them shows a statistically insignificant result, this current systematic review provides no further conclusion on whether photobiomodulation reduces pain in venous leg ulcers. No Adverse Events or side effects were reported, which is in concordance with how low fluences of photobiomodulation treatment are generally accepted as a safe. A RCT by Jagdeo et al. investigated the safety of red LED light (fluences of 160 J/cm^2^ to 640 J/cm^2^), here reporting cases of mild blistering, prolonged erythema and hyperpigmentation, but no serious adverse events [46]. Included studies in this review used fluences from 2.95 J/cm^2^ to 30 J/cm^2^, which explains why no adverse events have been reported related to the intervention.

The meta‐analysis from this systematic review indicates that photobiomodulation did not significantly improve ulcer area reduction compared with controls. The very high heterogeneity suggests considerable variability in treatment parameters, standard‐of‐care practices and methodological quality across trials. This may be attributed to the fact that many factors within photobiomodulation and wound healing still remain to be fully elucidated. For the future, the focus should be on consistent reporting of ulcer size measurements and variance data, providing the bare minimum for quantitative synthesis. Preclinical data are as heterogeneous as the clinical data, and the photobiomodulation field calls for expert guidelines in order to standardise photobiomodulation protocols with respect to wavelengths, fluences and treatment frequencies. However, photobiomodulation remains of clinical interest due to promising preclinical studies providing mechanistic evidence and its growing availability to the average consumer. Nevertheless, the current clinical evidence base is insufficient to recommend photobiomodulation as an effective adjunctive therapy for venous leg ulcers. Future well‐designed wound healing RCTs must adopt consistent treatment parameters and rigorous methodology to determine whether the biological potential of photobiomodulation translates into meaningful clinical outcomes.

## 5 Conclusion

This systematic review found no statistically significant improvement in venous leg ulcer healing with photobiomodulation compared with control treatment (MD 3.77 cm^2^; *p* = 0.37). Although photobiomodulation remains biologically plausible and appears safe at low fluences, current clinical evidence does not support its routine use as an adjuvant therapy for venous leg ulcers. High‐quality, adequately powered randomised trials with standardised photobiomodulation parameters are required to determine whether meaningful clinical benefits exist.

## Author Contributions

Design of the study: A.R., F.P., K.K.‐M., K.E.K. Conduction of search: A.R. and F.P. Selection of studies: A.R., F.P., and K.K.‐M. Data extraction: A.R. and F.P. Data analysis: A.R. and F.P. Writing of the first draft: A.R. Revising of the manuscript: A.R., F.P., K.K.‐M., K.E.K. Supervision: K.K.‐M. and K.E.K.

## Funding

The authors (A.R., F.P., K.K.‐M.) were supported by funding from Eurostars and Innovation Fund Denmark. The funders had no role in the conduct of the search strategy, data extraction, data analysis, or the writing process of the manuscript.

## Conflicts of Interest

The authors declare no conflicts of interest.

## Supporting information


**Table S1:** wrr70144‐sup‐0001‐TableS1.docx.

**Table S2:** All extracted wound healing outcomes.

## Data Availability

The data that support the findings of this study are available on request from the corresponding author. The data are not publicly available due to privacy or ethical restrictions.
