# Supplementary material for: The Healing Effect of Photobiomodulation on Venous Leg Ulcers: A Systematic Review and Meta‐Analysis
Source: Wound Repair Regen. 2026 Mar 26;34(2):e70144. doi: 10.1111/wrr.70144 (PMC13022061; doi:10.1111/wrr.70144)
Supplement: Supplementary file 1 — Table S1: wrr70144‐sup‐0001‐TableS1.docx. [file WRR-34-0-s001.docx]

| Author, Year |  | |  | | | Standard of care | | | | |
| --- | --- | --- | --- | --- | --- | --- | --- | --- | --- | --- |
|  | **Treatment per week** | **Cleansing** | | **Debridement** | **Topical Treatment** | | **Dressing** | **Compression** | **Oral systemic treatment** |  |
| Malm et al., 1991 | 2 | Saline | | NA | NA | | Paste Bandage | Diachlyon Bandage  (15-25 mmHg) | NA |  |
| Gupta et al., 1998 | 3 | Saline | | NA | NA | | Dry dressing | NA | NA |  |
| Franek et al., 2002 | 5 | NA | | NA | Potassium permanganate bath | | Wet dressings 0.1 % copper sulphate  + Compresses (Colistine, Fibrolan, Chlorampfenicol, Gentamicin) | Elastic bandages NS | NA |  |
| Lagan et al., 2002 | 1 | Water | | Sharp | NA | | NS | Compression +/- NS | NA |  |
| Kopera et al., 2005 | 3-7 | NA | | Enzymatic | Octenisept 2% | | Hydrofiber dressing | Compression NS | NA |  |
| Caetano et al., 2009 | 2 | Saline | | NA | Silver sulfadiazine 1% | | NS | Compression NS | NA |  |
| Leclére et al.,2010 | 1 | Saline | | Sharp | NA | | Hydrocelllular dressing | NA | NA |  |
| Taradaj et al., 2012 | 6 | NA | | NA | NA | | Sodium chloride 0,9 % dressing | NA | MPFF |  |
| Siqueira et al., 2014 | 1 | Saline | | Sharp | NA | | Zink oxide multilayer dressing | Inelastic compression (18-30 mmHg) | NA |  |
| Vitse et al., 2017 | 2 | NA | | Sharp | Hydrating gel | | NA | Support stockings  (30 mmHg) | NA |  |
| Pasek et al,. 2024 | 5 | NA | | Sharp | NA | | Hydrogel dressing | Compression  (34-46 mmHg) | Sulodexide, MPFF, Pentoxifylline  Acetylsalicylic acid |  |
