# Supplementary material for: The Healing Effect of Photobiomodulation on Venous Leg Ulcers: A Systematic Review and Meta‐Analysis
Source: Wound Repair Regen. 2026 Mar 26;34(2):e70144. doi: 10.1111/wrr.70144 (PMC13022061; doi:10.1111/wrr.70144)
Supplement: Supplementary file 2 — Table S2: All extracted wound healing outcomes. [file WRR-34-0-s002.docx]

| Author,  Year | Group | Mean or median ulcer area at baseline, cm^2^ ± SD / *cm^2^(Q1-Q3)* | Mean or median ulcer area at follow up, cm^2.^ ± SD/*cm^2^(Q1-Q3)* | | Absolute change in ulcer area, cm^2^ ± SD | Relative change in ulcer area, % | P-value on absolute difference baseline to follow up | P-value on relative difference baseline to follow up | P-value on absolute difference between groups | P-value on relative difference between groups |
| --- | --- | --- | --- | --- | --- | --- | --- | --- | --- | --- |
| Malm et al., 1991 | Light | 12.0 (4-52) | | NA | NA | NA | NA | NA | NA | NA |
|  | Control | 14.0 (3-44) | | NA | NA | NA | NA | NA |  |  |
| Gupta et al., 1998 | Light | 40.64±21.07 | | 21.34 | 19.3±7.04 | -47.49 ±NA | NA | NA | 0.0002 | NA |
|  | Control | 20.03±3.02 | | 18.56 | 1.47±5.12 | -7.34 ±NA | NA | NA |  |  |
| Franek et al, 2002 | Light | 15.76 | | 11.51 | 4.25 ± NA | -26.97 ± NA | 0.005 | NA | >0.05 | NA |
|  | Control | 13.25 | | 8.04 | 5.21 ± NA | -39.32 ± NA | 0.003 | NA |  |  |
| Lagan et al., 2002 | Light | NA | | NA | NA | -27.50 ±NA | NA | NA | NA | 0.14 |
|  | Control | NA | | NA | NA | -22.50 ±NA | NA | NA |  |  |
| *Kopera et al., 2005* | *Light* | *4.5 (1.2-36.7)* | | *4.5 (0.0-32.0)* | 0.00 ±NA | 0.00 ±NA | 0.683 | NA | NA | NA |
|  | *Control* | *8.60 (1.7-34.7)* | | *2.5 (1.5-18.5)* | 6.10 ±NA | -70.93 ±NA | 0.011 | NA |  |  |
| Caetano et al., 2009 | Light | 21.90±21.40 | | 10.95 ±NA | 10.95 ±NA | -50.00 ±0.22 | NA | NA | NA | <0.01 |
|  | Control | 15.20±9.20 | | 9.12 ±NA | 6.08 ±NA | -40.00 ±0.31 | NA | NA |  |  |
| Leclére et al.,2010 | Light | *2.83 (1.16-4.05)* | | 0.73 ± NA | 2.10 ± NA | -74.20 ±NA | NA | NA | NA | 0.60 |
|  | Control | *3.35 (1.82-8.3)* | | 0.19 ± NA | 3.16 ± NA | -94.30 ±NA | NA | NA |  |  |
| Taradaj et al., 2012 | Light | 17.92±12.02 | | 12.93 ± NA | 4.99 ± NA | -27.85 ±NA | NA | NA | NA | >0.05 |
|  | Control | 20.18±17.8 | | 14.76 ± NA | 5.42 ± NA | -26.88 ±NA | NA | NA |  |  |
| Siqueira et al., 2014 | Light | *8.00 (NA-15.0)* | | *2.00 (NA-)* | 6.00 ±NA | -75.00 ±NA | NA | NA | NA | >0.05 |
|  | Control | *7.00 (NA-14.0)* | | *20.0 (NA-)* | -13.00 ±NA | +85.71 ±NA | NA | NA |  |  |
| Vitse et al., 2017 | Light | 8.55±4.54 | | 2.28±2.78 | 6.27 ±3.66 | -77.10±25.7 | <0.0001 | NA | >0.8 | NA |
|  | Control | 9.93±6.57 | | 3.21±5.54 | 6.72 ±5.06 | -69.20 ±40.03 | 0.0013 | NA |  |  |
| Pasek et al., 2024 | Light | *3.75 (3.45-4.0)* | | *2.40 (1.95-2.9)* | 1.35 ± NA | -33.05 ±NA | <0.001 | NA | NA | <0.001 |
|  | Control | *3.60 (3.05-4.0)* | | *2.80 (2.60-3.1)* | 0.8 ± NA | -18.99 ± NA | <0.001 | NA |  |  |

Table S2. All extracted wound healing outcomes
